# Variations across Europe in hospitalization and management of pregnant women with SARS‐CoV‐2 during the initial phase of the pandemic: Multi‐national population‐based cohort study using the International Network of Obstetric Survey Systems (INOSS)

**DOI:** 10.1111/aogs.14643

**Published:** 2023-08-18

**Authors:** Odette de Bruin, Hilde Engjom, Nicola Vousden, Rema Ramakrishnan, Anna J. M. Aabakke, Outi Äyräs, Serena Donati, Eva Jónasdóttir, Marian Knight, Evelien M. Overtoom, Michele A. Salvatore, Miriam C. J. M. Sturkenboom, Teresia Svanvik, Reetta Varpula, An Vercoutere, Kitty W. M. Bloemenkamp, the INOSS COVID‐19 Working Group

**Affiliations:** ^1^ Department of Obstetrics, Birth Center Wilhelmina Children's Hospital, Division Woman and Baby University Medical Center Utrecht Utrecht the Netherlands; ^2^ Department of Biostatistics and Research Support, Julius Center for Health Sciences and Primary Care University Medical Center Utrecht Utrecht the Netherlands; ^3^ Division of Mental and Physical Health Norwegian Institute of Public Health Bergen Norway; ^4^ Department of Obstetrics and Gynecology Haukeland University Hospital Bergen Norway; ^5^ National Perinatal Epidemiology Unit, Nuffield Department of Population Health University of Oxford Oxford UK; ^6^ Department of Obstetrics and Gynecology Copenhagen University Hospital‐Holbæk Holbæk Denmark; ^7^ Department of Obstetrics and Gynecology Copenhagen University Hospital‐Nordsjælland‐Hillerød Hillerød Denmark; ^8^ Department of Obstetrics and Gynecology Helsinki University Hospital and University of Helsinki Helsinki Finland; ^9^ National Center for Disease Prevention and Health Promotion Istituto Superiore di Sanità – Italian National Institute of Health Rome Italy; ^10^ Department of Obstetrics and Gynecology Landspitali University Hospital Reykjavik Iceland; ^11^ Region Västra Götaland, Sahlgrenska University Hospital Department of Obstetrics and Gynecology Gothenburg Sweden; ^12^ Department of Obstetrics and Gynecology, CUB Hôpital Erasme Hôpital Universitaire de Bruxelles (H.U.B.), Université Libre de Bruxelles (ULB) Brussels Belgium

**Keywords:** COVID‐19, hospitalization, neonate, obstetric surveillance system, pregnancy, SARS‐CoV‐2

## Abstract

**Introduction:**

The majority of data on COVID‐19 in pregnancy are not from sound population‐based active surveillance systems.

**Material and methods:**

We conducted a multi‐national study of population‐based national or regional prospective cohorts using standardized definitions within the International Network of Obstetric Survey systems (INOSS). From a source population of women giving birth between March 1 and August 31, 2020, we included pregnant women admitted to hospital with a positive SARS‐CoV‐2 PCR test ≤7 days prior to or during admission and up to 2 days after birth. The admissions were further categorized as COVID‐19‐related or non‐COVID‐19‐related. The primary outcome of interest was incidence of COVID‐19‐related hospital admission. Secondary outcomes included severe maternal disease (ICU admission and mechanical ventilation) and COVID‐19‐directed medical treatment.

**Results:**

In a source population of 816 628 maternities, a total of 2338 pregnant women were admitted with SARS‐CoV‐2; among them 940 (40%) were COVID‐19‐related admissions. The pooled incidence estimate for COVID‐19‐related admission was 0.59 (95% confidence interval 0.27–1.02) per 1000 maternities, with notable heterogeneity across countries (*I* ^2^ = 97.3%, *P* = 0.00). In the COVID‐19 admission group, between 8% and 17% of the women were admitted to intensive care, and 5%–13% needed mechanical ventilation. Thromboprophylaxis was the most frequent treatment given during COVID‐19‐related admission (range 14%–55%). Among 908 infants born to women in the COVID‐19‐related admission group, 5 (0.6%) stillbirths were reported.

**Conclusions:**

During the initial months of the pandemic, we found substantial variations in incidence of COVID‐19‐related admissions in nine European countries. Few pregnant women received COVID‐19‐directed medical treatment. Several barriers to rapid surveillance were identified. Investment in robust surveillance should be prioritized to prepare for future pandemics.

## Graphical abstract

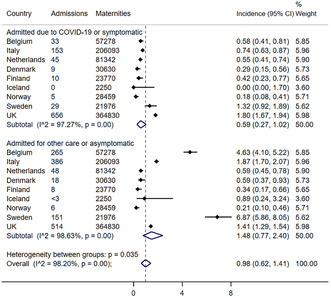

**Abbreviations**
BMI: body mass index
CI: confidence interval
COVID‐19: coronavirus disease
ICU: intensive care unit
INOSS: International Network of Obstetric Survey systems
PCR: polymerase chain reaction
SARS‐CoV‐2: severe acute respiratory syndrome coronavirus 2

**Key message**

Across Europe, substantial variation in COVID‐19‐related admission and clinical management in pregnant women was observed. This may reflect different national public health strategies early in the pandemic and emphasizes the need for alignment of management and treatment recommendations globally.

## 1 INTRODUCTION

In 2021, a World Health Organization (WHO) systematic review concluded that SARS‐CoV‐2 infection in pregnancy was associated with an increased risk of admission to an intensive care unit (ICU) for the mother, as well as preterm birth, and admission to a neonatal unit for the infant. Maternal age over 35 years, high body mass index (BMI), non‐white ethnicity, preexisting diabetes, chronic hypertension and preeclampsia were associated with severe COVID‐19 in pregnancy, although the definition of severe disease was not always clear.^1^ Another systematic review on medical treatment reported that only few pregnant women received medication for COVID‐19.^2^ However, the majority of included studies were conducted outside of Europe, most were hospital‐based studies or case series, and only a small number of women with severe outcomes were included. Most studies were unclear regarding the timing and reason for the SARS‐CoV‐2 test and hospital admission. Additional issues encountered in these systematic reviews were the variation in the data items collected and the definitions used.^1^, ^2^

Whereas several ad‐hoc case‐based data collections were set up, there was a lack of population‐based studies using standardized definitions of exposures and outcomes. Countries participating in the International Network of Obstetric Survey Systems (INOSS) collaboration conduct national or regional population‐based surveillance studies of severe complications or rare diseases in pregnancy.^3^ The INOSS collaboration was established in 2010, and later developed common protocols with uniform definitions and core data items.^4^ The network has previously been used to rapidly collect information to inform policymakers and offer guidance in pandemics and other emerging infections.^5^, ^6^, ^7^

These experiences enabled the network to swiftly commence standardized national or regional studies in response to the SARS‐CoV‐2 pandemic.^8^, ^9^, ^10^, ^11^, ^12^, ^13^, ^14^, ^15^ The main aim of this study was to assess incidence of hospitalization with SARS‐CoV‐2 infection among pregnant women, across multiple European countries, during the first months of the pandemic. A secondary aim was to describe the COVID‐19‐directed medical treatment of these women hospitalized with SARS‐CoV‐2.

## 2 MATERIAL AND METHODS

This was a multi‐national population‐based study within the INOSS (EUPAS40489). The INOSS countries collect ongoing prospective data on maternal mortality, severe maternal morbidity and rare diseases in pregnancy. As the obstetric surveillance systems are ongoing, topics can be swiftly introduced. Therefore, at the start of the pandemic, most countries had national permission in place to start retrieving clinical data promptly. After discussions within INOSS, the national and regional population‐based cohort studies were used to collect individual information about pregnant women with SARS‐CoV‐2 admitted to hospital in Belgium (BE), Italy (IT), the Netherlands (NL), United Kingdom (UK), Denmark (DK), Finland (FI), Iceland (IS), Norway (NO) and Sweden (SE).^8^, ^9^, ^10^, ^11^, ^12^, ^13^, ^14^, ^15^

In each participating country, standardized case report forms were used to retrieve prospectively recorded information from clinical records. Cases were identified by reporting clinicians or by combining data from perinatal/birth registries, and hospital discharge records. National quality control by linkage to other data sources were performed if possible; this process is summarized by country in Appendix S2.

The source population comprised all pregnant women giving birth in participating countries and regions between March 1, 2020 and August 31, 2020. The estimated total number of maternities was based on notified births to national perinatal/birth registries systems if available, or alternatively on hospital data.^3^ Women were included if they were admitted to hospital with a positive SARS‐CoV‐2 polymerase chain reaction (PCR) test within 7 days prior to or during admission in pregnancy, and up to 2 days after giving birth. This definition did not distinguish between different indications for SARS‐CoV‐2 testing. To discriminate between the symptomatic women who needed COVID‐19‐related healthcare and pregnant women who were screened at admission for labor and obstetric care, admission was categorized as COVID‐19‐related or non‐COVID‐19‐related. If the cause of admission was unknown, women were classified as COVID‐19‐related if they were reported to have any symptoms and as non‐COVID‐19 if reported to be asymptomatic. Women who were discharged with ongoing pregnancies were followed up until they gave birth.

The primary outcome was the incidence of admission with a SARS‐CoV‐2 infection per 1000 maternities. Secondary outcomes included severe maternal and perinatal outcomes. The severe maternal outcomes were maternal death (COVID‐19‐related during admission), maternal ICU admission due to COVID‐19, and maximum level of respiratory support (mechanic ventilation or extracorporeal membrane oxygenation [ECMO], continuous positive airway pressure [CPAP] or high‐flow nasal cannula, and oxygen supplementation). COVID‐19‐directed medication included: antibiotics, antivirals, hydroxychloroquine, anti‐interleukin 6, intravenous (i.v.) immunoglobulins, corticosteroids for fetal or maternal indication, and low molecular weight heparin (LMWH) for thromboprophylaxis or treatment of thromboembolic disease. Severe neonatal outcomes included stillbirth (intrauterine death prior to or during labor after 22 weeks’ gestation, or after 24 weeks’ gestation in the UK), admission to neonatal unit, and neonatal death (before discharge following birth). These neonatal outcomes were assessed with the infant as the unit of analysis to account for multiple pregnancies. Pregnancy loss and termination of pregnancy were not addressed in this study.

The covariates included maternal age at positive SARS‐CoV‐2 test, body mass index (BMI, pre‐pregnancy weight or earliest recorded weight in pregnancy), obesity (BMI ≥30), migrant or minority background, parity (previous births after 22 weeks’ gestation or 24 weeks in the UK), gestational age at infection and at birth (based on last menstrual period or ultrasound according to national guidelines) and mode of birth (vaginal birth or cesarean section). Migrant background was defined as maternal country of birth outside of Europe. Minority background was defined as black, Asian, Chinese, Mixed, or other ethnic minorities (BAME).

### 2.1 Statistical analyses

A random effects model was used to pool national incidence using Freeman–Tukey double arcsine transformation, and Wilson's method for confidence intervals.^16^, ^17^, ^18^ The incidence of hospitalization per 1000 maternities was reported by admission group with 95% confidence intervals (CI) for each country separately. Heterogeneity across studies was assessed using the *I* ^2^ statistic.

Secondary outcomes were rare and consequently pooling for further analyses was not feasible. The secondary outcomes are described by country to show the variation in characteristics across countries. Proportions are presented as percentages with the range across countries. If information about risk factor or outcome was missing, the analysis was performed based on the total number with complete information. Numbers of missing data are presented in Tables S2–S4.

National analyses were performed using IBM SPSS Statistics (IBM Corp.) or STATA (STATA Corp. LLC). Aggregate national results were analyzed using STATA.

### 2.2 Ethics statement

This study was evaluated by the medical review committee at University Medical Center, Utrecht, and was deemed exempt from ethical review due to the use of anonymous national level data (protocol no. 21/682, October 4, 2021). All relevant guidelines have been followed and necessary ethics committee approvals have been obtained according to national regulations in each INOSS country (Appendix S3). Data‐sharing agreements for anonymous, aggregated data were signed if required with participating countries. Data were managed and stored in accordance with national regulations and the General Data Protection Regulation. Numbers from national datasets from Denmark, Finland, Iceland and Norway were merged to avoid reporting of small numbers.

## 3 RESULTS

**FIGURE 1:**
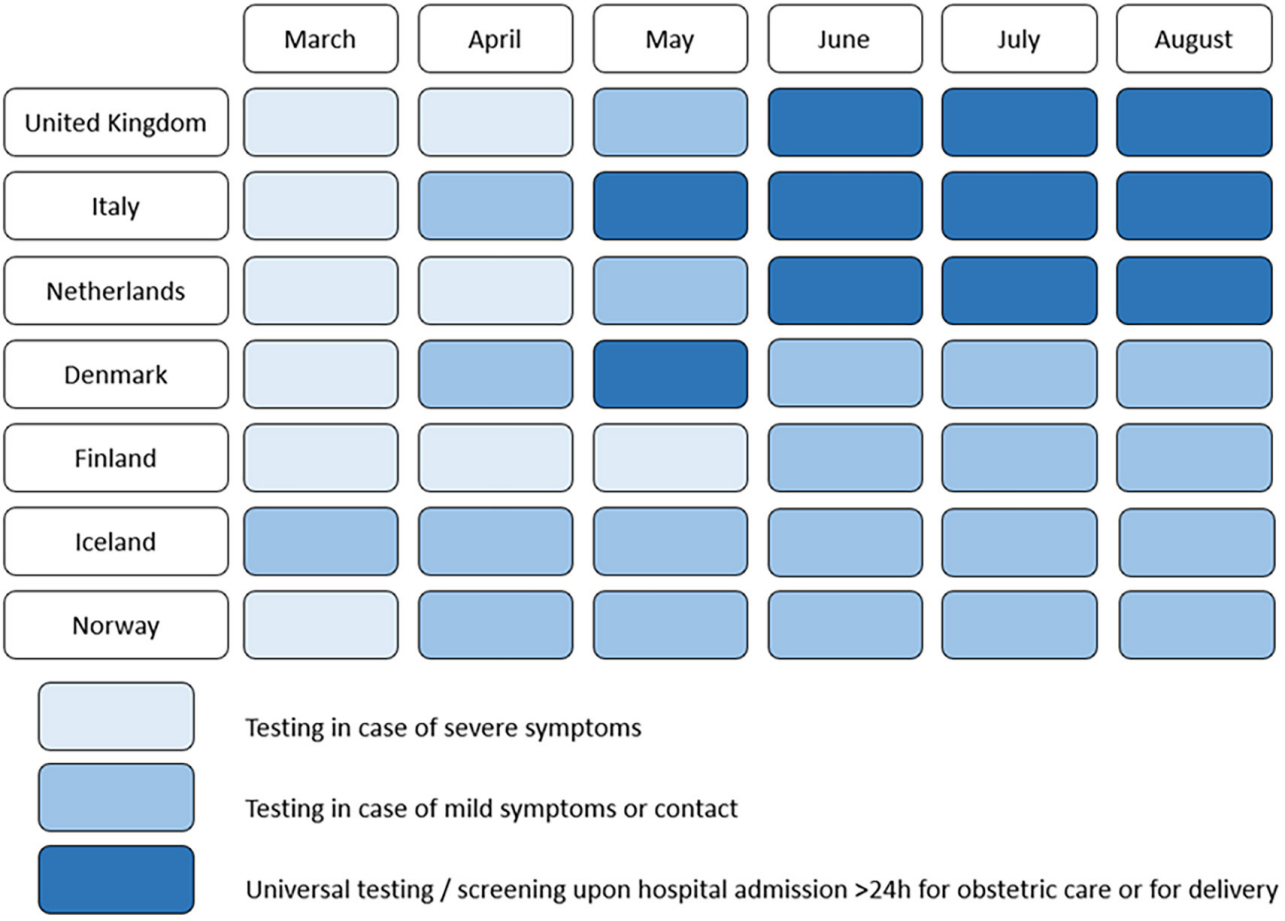
Overview of SARS‐CoV‐2 testing strategies in pregnancy in the seven European countries from March to August 2020. In Belgium there were no national testing strategies and testing strategies differed across hospitals and regions. Similarly, testing strategies differed across hospitals and regions in Sweden, with early implementation of universal testing in Stockholm.

The estimated number of maternities captured by the pregnancy surveillance systems from March 1, 2020 and August 31, 2020 were 816 628. In this source population, 2338 pregnant women had a positive SARS‐CoV‐2 PCR test and were admitted to hospital (Figure S1). The testing strategies varied in the different areas, and Figure 1 indicates the approximate timing of testing strategy changes in each of the participating countries. Across all countries and the whole study period, symptomatic women admitted to hospital were tested. However, screening procedures varied between countries, from no screening during the whole period, to early implementation of routine testing of asymptomatic women admitted for labor or obstetric care.

Of the 2338 pregnant women, 940 (40.2%) had COVID‐19‐related admissions, whereas 1398 (59.8%) tested positive for SARS‐CoV‐2 but were admitted for non‐COVID‐19 healthcare, most frequently labor or obstetric care (Figure S1). Table 1 shows the characteristics of the study population by admission group in each country. In most countries, the number of women with risk factors was low and pooled assessment of risk factors was not feasible. In all countries and regions, most women in the COVID‐19 admission group were in the third trimester of pregnancy.

**TABLE 1:** Characteristics and birth outcomes of pregnant women with a positive SARS‐CoV‐2 PCR test within 7 days prior to admission and up to 2 days after giving birth from March to August 2020 by cause of admission and country.

	BOSS	ItOSS	NethOSS	NOSS		UKOSS
	National, Belgium	National, Italy	National, the Netherlands	National, Denmark, Finland, Iceland & Norway	Regional, Sweden	National, United Kingdom
Hospital admission, *n*						
COVID‐19‐related admissions	33	153	45	24	29	656
Non‐COVID‐19‐related admissions	265	386	48	32	151	514
Age ≥35 years, *n* (%)						
COVID‐19‐related admissions	8 (24.2)	55 (35.9)	11 (24.4)	7 (29.2)	11 (37.9)	226 (34.5)
Non‐COVID‐19‐related admissions	53 (20.0)	119 (31.4)	16 (33.3)	9 (28.1)	51 (33.8)	130 (25.3)
BMI >30 kg/m^2^, *n* (%)						
COVID‐19‐related admissions	7 (21.9)	30 (19.9)^a^	11 (28.9)	8 (33.3)	9 (34.6)	213 (33.6)
Non‐COVID‐19‐related admissions	48 (19.5)	33 (8.7)^a^	15 (34.9)	5 (15.6)	32 (21.2)	129 (25.8)
Migrant background or BAME ethnicity, *n* (%)						
COVID‐19‐related admissions	NA	57 (37.3)	19 (47.5)	11 (45.8)	22 (78.6)	364 (56.1)
Non‐COVID‐19‐related admissions	NA	101 (26.2)	16 (34.0)	14 (45.2)	84 (56.8)	179 (35.5)
Multiparous, *n* (%)						
COVID‐19‐related admissions	23 (69.7)	101 (66.4)	22 (48.9)	15 (62.5)	21 (77.8)	396 (60.6)
Non‐COVID‐19‐related admissions	184 (69.4)	203 (52.7)	29 (60.4)	21 (65.6)	96 (63.6)	284 (56.0)
Gestational age at infection						
First trimester, *n* (%)						
COVID‐19‐related admissions	0	8 (5.3)	1 (2.2)	3 (13.0)	0	23 (3.5)
Non‐COVID‐19‐related admissions	2 (0.8)	2 (0.5)	2 (4.2)	1 (3.1)	0	14 (2.7)
Second trimester, *n* (%)						
COVID‐19‐related admissions	12 (36.4)	24 (15.8)	13 (28.9)	6 (26.1)	5 (20.8)	107 (16.3)
Non‐COVID‐19‐related admissions	6 (2.3)	7 (1.9)	1 (2.1)	3 (9.4)	7 (4.7)	34 (6.6)
Third trimester, *n* (%)						
COVID‐19‐related admissions	21 (63.6)	120 (78.9)	31 (68.9)	14 (60.9)	21 (87.5)	526 (80.2)
Non‐COVID‐19‐related admissions	257 (97.0)	365 (97.6)	45 (93.8)	28 (87.5)	141 (95.3)	466 (90.7)
Cesarean section, *n* (%)						
COVID‐19‐related admissions	8 (24.2)	53 (46.1)	15 (34.9)	11 (47.8)	9 (33.3)	313 (47.7)
Non‐COVID‐19‐relatedadmissions	45 (17.0)	113 (30.7)	15 (31.3)	4 (18.2)	43 (28.7)	213 (42.7)
Preterm birth (<37 weeks), *n* (%)						
COVID‐19‐related admissions	1 (3.0)	20 (17.4)	8 (18.6)	5 (21.7)	3 (12.5)	136 (20.7)
Non‐COVID‐19‐related admissions	20 (7.6)	41 (11.5)	6 (13.3)	1 (3.3)	23 (15.5)	64 (12.7)

Abbreviations: BAME, black, Asian and minority ethnic; BMI, body mass index; COVID‐19, coronavirus disease 2019; NA, not available.

^a^ These cases were reported with a check‐box obesity (yes/no), resulting in fewer cases missing than for numerical BMI value.

**FIGURE 2:**
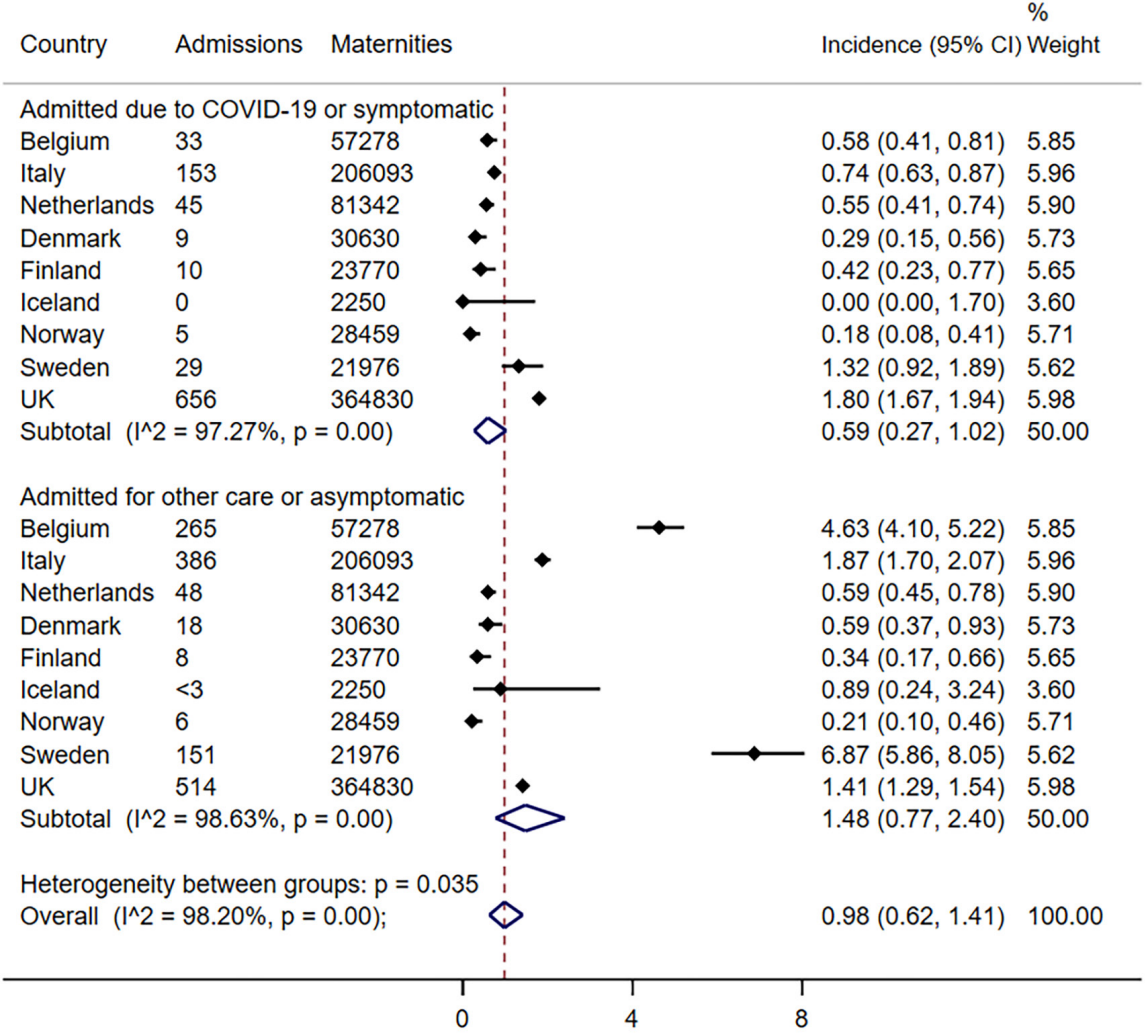
Pooled risk of hospitalization per 1000 maternities by cause of admission.

The overall pooled incidence estimate for hospitalization for any cause among SARS‐CoV‐2‐positive pregnant women was 0.98 (95% confidence interval [CI] 0.62–1.41) per 1000 maternities across Europe. The pooled incidence estimate for COVID‐19‐related admission was 0.59 (95% CI 0.27–1.02) per 1000 maternities, ranging from no admissions in Iceland to 1.8 per 1000 maternities in the UK (Figure 2). There was notable heterogeneity across countries (*I* ^2^ = 97.3%, *P* = 0.00). The incidence of non‐COVID‐related admission varied even more widely, with similarly high heterogeneity (*I* ^2^ = 98.6%, *P* = 0.00) (Figure 2), and was likely to be influenced by screening policies.

**FIGURE 3:**
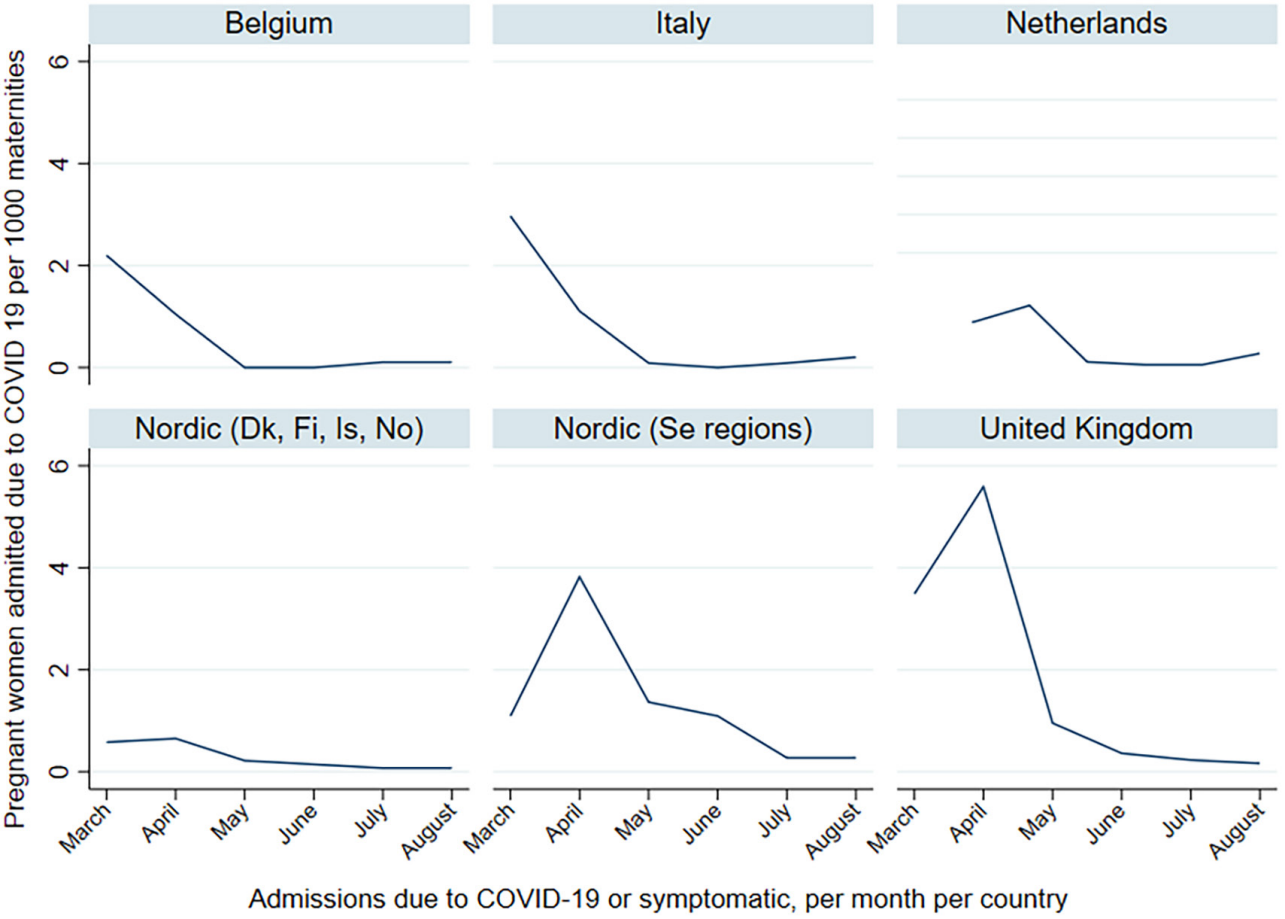
Admission due to COVID‐19 per 1000 maternities per month of first positive PCR test, March to August 2020, by country.

Figure 3 shows the incidence of COVID‐19‐related admission per 1000 maternities by month of first positive test. In Belgium and Italy, the hospitalization rate was highest in March 2020. There was initially an increase in incidence in the Netherlands, Sweden and the UK, showing a peak in April 2020. The incidence of COVID‐19‐related hospitalization in the Nordic countries Denmark, Finland and Norway was low between March and August 2020. The incidence of admission per 1000 maternities by month of first positive test, stratified by reason of admission, is presented in Figure S2.

Severe maternal outcomes and COVID‐19‐directed medical treatment in the COVID‐19‐related admission group is shown in Table 2. ICU admission ranged from 8% to 17% across countries and 5%–13% needed mechanical ventilation or ECMO. Seven maternal deaths related to COVID‐19 were reported. Antibiotics alone for any indication were prescribed more frequently in Belgium (49%), the Netherlands (44%) and the Nordic countries (41%) than in the UK (27%) and Italy (7%). Twelve women, 0%–3% across countries, received antiviral medication. Hydroxychloroquine was prescribed more frequently in Italy (58%) and Belgium (45%) than in the Netherlands (11%); there were no prescriptions in the Nordic countries and the UK. Administration of corticosteroids for maternal indication varied from no prescriptions in the Netherlands to a few prescriptions in the other countries. Thromboprophylaxis was administered less frequently in the UK (14%) than in Belgium (36%), Italy (36%) and the Nordic countries (55%). Treatment of thromboembolism was rare, with only three prescriptions in total.

**TABLE 2:** Medical treatment and maternal outcomes among pregnant women admitted due to COVID‐19 from March to August 2020 by country.

	BOSS	ItOSS	NethOSS	NOSS	UKOSS
	National, Belgium	National, Italy	National, the Netherlands	National, Denmark, Finland & Norway	National, United Kingdom
Total women admitted due to COVID‐19, *n*	33	153	45	24	656
Medical treatment, *n* (%)					
Antibiotics (alone)	16 (48.5)	11 (7.2)	20 (44.4)	9 (40.9)	174 (26.5)
Antivirals (alone)	1 (3.0)	4 (2.6)	0	0	7 (1.1)
Antibiotic and antiviral in combination	0	9 (5.9)	2 (4.4)	4 (18.2)	8 (1.2)
Hydroxychloroquine	15 (45.5)	88 (57.9)	5 (11.1)	0	0
Anti‐Interleukin 6	0	NA	0	0	0
I.v. Immunoglobulins	0	NA	NA	0	0
Steroids for fetal indication	3 (9.1)	21 (13.8)	6 (13.3)	6 (27.3)	105 (16.0)
Steroids for maternal indication	3 (9.1)	NA	0	2 (9.1)^b^	7 (1.1)
Thrombosis prophylaxis	12 (36.4)	55 (36.2)	NA	12 (54.5)	89 (13.6)
Anti‐thrombotic treatment	1 (3.0)	NA	NA	1 (4.5)	1 (0.2)
ICU admission, *n* (%)	3 (9.1)	15 (9.8)	4 (8.9)	4 (17.4)	53 (8.1)
Maximum level of respiratory support, *n* (%)^a^					
Mechanical ventilation or ECMO	4 (12.1)	7 (4.6)	3 (6.7)	3 (13.0)	25 (13.2)
CPAP or high flow nasal cannula	NA	19 (12.4)	NA	0	15 (7.9)
Oxygen supplementation	NA	44 (28.8)	22 (48.9)	6 (28.6)	17 (8.9)
Maternal death, *n* (%)	0	0	0	0	7 (1.1)

Abbreviations: COVID‐19, coronavirus disease 2019; CPAP, continuous positive airway pressure; ECMO, extracorporeal membrane oxygenation; ICU, intensive care unit; I.v., intravenous; NA, not available.

^a^ In UKOSS, maximum level of respiratory support was measured only among women who received respiratory support (*n* = 190); level of respiratory support was unknown for 57/190 women.

^b^ Includes one with known non‐COVID‐19 indication.

Women with COVID‐19‐related admission gave birth to 908 infants with available information, including 5 (0.6%) stillbirths. Among the live‐born infants, 14%–30% across countries were admitted to neonatal unit, and 2 (0.2%) neonatal deaths were reported (Table S1).

## 4 DISCUSSION

In this multi‐national study, using population‐based cohorts from nine European countries or regions during the first months of the pandemic, we found substantial variations in the incidence of COVID‐19‐related admissions. Additionally, the low number of women who received COVID‐19‐directed medical treatment was striking.

To the best of our knowledge, this is the largest multi‐national dataset containing population‐level data on pregnant women with SARS‐CoV‐2. The national INOSS studies took into account more than 800 000 women giving birth. The population‐based nature provided estimates of incidence, and in comparison with hospital or case‐based studies, these estimates are less vulnerable to bias such as selection and reporting. The clinical information was retrieved from medical records and enabled discrimination between asymptomatic and symptomatic women. This reduced the risk of misclassification bias, attributing severe outcomes to COVID‐19 rather than to obstetric complications, and enabled comparison across countries with different testing policies. Still, the testing policies varied over time, and stratification of the analyses by COVID‐19‐related admission may not fully account for this heterogeneity in testing. The varying testing strategies also affected the incidence of infection in the general population across Europe.^19^

The use of a comparator group of pregnant women admitted to hospital for non‐COVID‐19‐related reasons is a limitation of this study. Data on pregnant women who were not admitted to hospital as a comparator group would allow further exploration of the consequences of mild/asymptomatic infection. Such data are available in some of the INOSS countries but are not included in the present study.^11^, ^15^ Additionally, the use of uniform case report forms with similar definitions aided comparability across countries; however, it was not possible to compile completely uniform datasets.

Although the INOSS collaboration enabled collection of real‐time data during the pandemic readily, analysis and reporting of the data was delayed by the lack of funding and staff. The collaboration between clinicians and epidemiologists was key to the relevance of the study information, but as the pandemic progressed, clinical workloads increased. Alongside this, even though the countries planned national quality control by linkage to other national registries, not all countries have yet been able to assess completeness at the time of analysis. We observed a substantial lag time in availability of registry data in some countries and further delays due to the pandemic have also been described for publication of European routine perinatal data.^20^

The difference in admission rate due to COVID‐19 between countries may be related to different public health strategies to contain and limit viral transmission as well as other factors including population density, social security and public trust. The multi‐national approach allowed for comparison of the impact of varying timing and nature of public health measures implemented in the countries. According to the Oxford Coronavirus Government Response tracker, strict national public health measures were implemented from the beginning of the pandemic in Italy, which coincided with the decline in the hospitalization rate from March 2020.^21^ The public health measures in the UK, the Netherlands and Sweden were less strict at the beginning and were enforced later, which may explain the peak in hospitalization rates in April 2020.^21^ This may provide evidence to confirm that the timing and nature of public health measures does have an impact on the risk of infection and thus also on the risk of COVID‐19‐related admission among pregnant women. This knowledge could aid consideration of measures for future SARS‐CoV‐2 waves or emerging infections, especially in a population that is reluctant to vaccinate.^22^

Furthermore, because of the large variation in incidence of COVID‐19‐related admission and severe disease, the current dataset had too few severe events in most countries to assess risk factors reliably. Despite this, we confirm overall high frequencies of previously described risk factors associated with COVID‐19.^1^ Some of the individual INOSS countries were able to compare the characteristics among pregnant women with COVID‐19‐related admissions with appropriate control groups or population data; they have reported age ≥35 years, BMI, migrant background or BAME, preterm birth, cesarean section and neonatal unit admission to be associated with admission for COVID‐19‐related healthcare in pregnancy.^8^, ^9^, ^10^, ^11^, ^12^, ^13^, ^14^, ^15^ However, the number of severe events in most of the countries was low during the first wave, with most ICU admissions in Italy and the UK. Population‐level data from six Canadian provinces for the period from March 2020 to October 2021 showed similar findings, indicating that higher age, advanced gestational age and preexisting hypertension were significantly associated with poorer maternal outcomes. COVID‐19‐affected pregnancies also demonstrated a significantly increased risk of preterm birth. Consistent with our study, non‐COVID‐19‐related admissions were excluded from their analyses.^23^

Variation in the clinical management between countries may arise from different thresholds for interventions for COVID‐19, as well as for obstetric complications. ICU admission may vary depending on local capacity, experience and national medical practice and guidelines, and it is important to discriminate between COVID‐19 and non‐COVID causes related to other obstetric emergencies.^24^ Furthermore, early in the pandemic, knowledge was lacking about the effect of COVID‐19 in pregnancy, and safety data of COVID‐19 medicines in pregnant women were not yet available. As a result, national treatment guidelines varied and treatment might have depended on the discretion of the clinician or institution.

From April 2020 there was a conditional recommendation for prescription of thromboprophylaxis in Belgium, the Netherlands and the UK, and a strong recommendation in Italy and Norway.^25^, ^26^, ^27^, ^28^, ^29^ Despite this, only a small number received thromboprophylaxis. The low levels observed may have been caused by concerns about the association between COVID‐19 and thrombocytopenia.^27^ The low utilization of corticosteroids for maternal indication could reflect the concern for disease progression when using steroids.^30^ In June 2020, the RECOVERY trial demonstrated that the use of corticosteroids was effective in reducing mortality and the duration of invasive ventilation.^31^ Following that, it was implemented in national guidelines.^26^, ^27^, ^29^ Likewise, the use of hydroxychloroquine varied between countries, with frequent prescription during the initial months of the pandemic in Italy and Belgium. These prescriptions were based on available safety data without evidence of efficacy, mostly within a research setting.^32^, ^33^ In June 2020, the RECOVERY trial demonstrated that it was not effective and guidance against use of these treatment was issued.^34^ We also observed large variation in the use of antibiotics between countries. However, the indication for antibiotic use was unknown, and therefore they may have been given for other reasons. The overall utilization of COVID‐19 medical treatments during the first months was low, consistent with the review of Giesbers et al.^2^ Pregnant women are a vulnerable population that, with the exception of the RECOVERY trial, is often excluded from large international trials and early phase research on medicines and vaccines, contrary to pre‐pandemic policy guidance.^31^, ^34^, ^35^, ^36^

To better prepare for a future pandemic, it is crucial to hibernate study protocols with all necessary approvals and allocated funding in place, so that research can quickly resume when needed. Additionally, enabling rapid registry linkages and prioritizing robust population‐based registry information on the burden of infection and core maternal and perinatal outcomes can help healthcare systems respond more effectively to future pandemics.

## 5 CONCLUSION

The INOSS showed variation between the participating European countries in the incidence of COVID‐19‐related admission among pregnant women and in their clinical management of the disease, during the first months of the pandemic. Population‐based surveillance systems are useful during pandemics to provide real‐time data on the management and outcomes of infection in pregnant women, which directly guide healthcare workers and politicians on how to react. Hence, there is an urgent need to invest robust funding in infrastructure and capacity to monitor the impact of emerging infections on pregnant women and their babies.

Few pregnant women received medical treatment targeted at the SARS‐CoV‐2 infection during the first months. This may have resulted in the under‐treatment of severely ill pregnant women. While caution is warranted, medical treatment should not be withheld due to pregnancy. Pregnant women should be included in the development, and trials, of medications and vaccines. A planned individual data meta‐analysis with longer follow‐up within INOSS will give more insight into the effect of factors and medications associated with better outcomes.

## AUTHOR CONTRIBUTIONS

All authors contributed to planning and analysis of the national surveillance within the INOSS network and the conception of the international study. HE, NV, RR, AJMA, OÄ, SD, EJ, MK, EMO, MAS, TS, RV, AV, and KWMB had the main responsibility for the respective national datasets. OdB, HE, NV, RR, MCJMS and KWMB drafted the protocol for the international study, coordinated the compilation of national data, and drafted the paper based on discussions with all authors and the scientific committee (SD, MK and KWMB). All authors participated in revision of the paper and the decision to submit, approved the final version and accept responsibility for the article as published.

## FUNDING INFORMATION

The national studies reported the following funding sources: The BOSS project was funded by the Belgian Federal Public Service of Health. The NOSS collaboration was supported by the Nordic Federation of Societies of Obstetrics and Gynecology (grant no. 6505, 2020). NOSS‐Denmark was supported by grants from The Region of Southern Denmark and Region Zealand's shared fund for joint health research projects (Reg. no. A767), and EasyTrial provided the data collection software. NOSS‐Finland received grants from the Finnish Medical Society, and from Helsinki University. UKOSS received funding from the National Institute for Health Research HS&DR Program (11/46/12). The national studies in Italy and the Netherlands did not have specific funding. The multi‐national study received partial funding support from the European Medicines Agency (EMA) under the Framework service contract nr EMA/2018/28/PE. The content of this paper expresses the opinions of the authors and may not be understood or quoted as being made on behalf of or reflecting the position of the EMA or any of its committees or working parties. The research leading to these results was conducted as part of the activities of the EU PE&PV (Pharmacoepidemiology and Pharmacovigilance) Research Network, which is a public academic partnership coordinated by Utrecht University, the Netherlands. The CONSIGN project was scientifically coordinated by the University Medical Center, Utrecht.

## CONFLICT OF INTEREST STATEMENT

OB declares support from the European Medicines agency (EMA). HE declares grants from the Nordic Federation of Societies of Obstetrics and Gynecology (NFOG) and Norwegian Research Council (grant no 320181). AA declares a grant from the Region of Southern Denmark and Region Zealand's shared fund for joint health research projects. OA declares grants from the Finnish Medical Association and NFOG. MK declares grants from the National Institute for Health and Care Research, Medical Research Council, Healthcare Quality Improvement Partnership and Wellbeing of Women during the course of the study. MS leads a department that conducts studies on COVID‐19 vaccines for the European Medicines Agency, Pfizer, AstraZeneca and Janssen. All support was according to the ENCePP code of conduct. None of the other authors (NV, RR, SD, EJ, EO, MAS, TS, RV, AV, KB) has anything to disclose.

## Supporting information


Appendix S1.

Appendix S2.

Appendix S3.

Table S1.
Table S2.
Table S3.
Table S4.
Figure S1.
Figure S2.
