# Supplementary material for: Variations across Europe in hospitalization and management of pregnant women with SARS‐CoV‐2 during the initial phase of the pandemic: Multi‐national population‐based cohort study using the International Network of Obstetric Survey Systems (INOSS)
Source: Acta Obstet Gynecol Scand. 2023 Aug 18;102(11):1521–30. doi: 10.1111/aogs.14643 (PMC10577630; doi:10.1111/aogs.14643)
Supplement: Supplementary file 1 — Appendix S1. [file AOGS-102-1521-s002.docx]

**Appendix 1: Members of the INOSS COVID-19 in pregnancy working group**

Mika Gissler, Department of Knowledge Brokers, THL Finnish Institute for Health and Welfare, Helsinki, Finland. ORCID 0000-0001-8254-7525.
Karin Källén, Centre of Reproduction Epidemiology, Lund University, Lund, Sweden. ORCID 0000-0001-5765-2630.
Karin Pettersson, Division of Obstetrics and Gynecology, Department of Clinical Science, Intervention and Technology, Karolinska Institute and Karolinska University Hospital. ORCID 0000-0001-6549-504.
Lars Thurn, Department of Obstetrics and Gynecology, Skåne University Hospital, Lund. ORCID 0000-0001-9813-029X.
Elin Jones, Department of Obstetrics and Gynecology, Karolinska University Hospital, Stockholm, Sweden. ORCID 0000-0003-2610-1849.
Julia Kanerva, Department of Obstetrics and Gynecology, Institute of Clinical Sciences, Sahlgrenska Academy, University of Gothenburg, Gothenburg, Sweden.
Kari Klungsøyr, Division of Mental and Physical Health, Norwegian Institute of Public Health and Department of Global Public health and Primary Care, University of Bergen, Norway. ORCID 0000-0003-2482-1690.
Lill-Trine Nyfløt, Department of Obstetrics, Vestre Viken Hospital Drammen and the Norwegian Research Centre for Womens’s Health, Oslo University Hospital, Oslo, Norway. ORCID 0000-0001-8979-1563.
Siri Vangen, Norwegian Research Centre for Womens’s Health, Oslo University Hospital, Oslo, Norway. ORCID 0000-0003-4681-4774.
Lone Krebs, Department of Obstetrics and Gynecology, Copenhagen University Hospital – Amager and Hvidovre, Hvidovre, Denmark and Department of Clinical Medicine, University of Copenhagen, Copenhagen, Denmark. ORCID 0000-0001-5433-4776.
Thomas van den Akker, Department of Obstetrics and Gynecology, Leiden University Medical Center, Leiden, the Netherlands and Athena Institute, Vrije Universiteit Amsterdam, Amsterdam, the Netherlands. ORCID 0000-0002-9890-9145.
Karolien Benoit, Department of Obstetrics and Gynaecology, Ghent University Hospital, Belgium.
Griet Vandenberghe, Department of Obstetrics and Gynaecology, Ghent University Hospital, Belgium
Kristien Roelens, Department of Obstetrics and Gynaecology, Ghent University Hospital, Belgium
Edoardo Corsi Decenti National Centre for Disease Prevention and Health Promotion, Istituto Superiore di Sanità – Italian National Institute of Health, Rome, Italy
Catherine Denaux, Université Paris Cité, CRESS UMR 1153, Obstetrical Perinatal and Paediatric Epidemiology Research Team, EPOPé, INSERM, INRAE, Paris, France
Aurelien Seco, Université Paris Cité, CRESS UMR 1153, Obstetrical Perinatal and Paediatric Epidemiology Research Team, EPOPé, INSERM, INRAE, Paris, France
Montse Palacio, Institut Clínic de Ginecologia, Obstetrícia i Neonatologia, Hospital Clínic, Universitat de Barcelona, Barcelona, Spain. ORCID 0000-0002-5869-4629.
