# Supplementary material for: Variations across Europe in hospitalization and management of pregnant women with SARS‐CoV‐2 during the initial phase of the pandemic: Multi‐national population‐based cohort study using the International Network of Obstetric Survey Systems (INOSS)
Source: Acta Obstet Gynecol Scand. 2023 Aug 18;102(11):1521–30. doi: 10.1111/aogs.14643 (PMC10577630; doi:10.1111/aogs.14643)
Supplement: Supplementary file 2 — Appendix S2. [file AOGS-102-1521-s003.docx]

**Appendix 2: Data quality verification**

Belgium

The Belgium Obstetric Surveillance System (B.OSS) collected information about admission to hospital for women with COVID-19 infection in pregnancy and up to 42 days postpartum.^1^ Cases were notified by reporting clinicians at each hospital. Enrolment was closed at the end of February 2021. Enrolled pregnancies were followed till the end of pregnancy. National perinatal statistics for 2020 were available in December 2021 as denominator data. BOSS data could not be linked to hospital discharge databases because it was not possible to obtain agreement with the Committee of Security Information. BOSS data was linked to perinatal registries, and denominator perinatal data were available nationwide, but separately for Flanders, Brussels and Wallonia.

Italy

The Italian Obstetric Surveillance System (ItOSS) collected national data on hospital admissions of women with SARS-CoV-2 infection in pregnancy and up to 42 days postpartum.^2^ ItOSS sent out weekly notification reminders to identify new cases and contacted clinicians by email and telephone in case of zero reporting. The completeness of the data collection forms was verified monthly, and clinicians were requested to complete missing data. Maternal deaths were cross checked with the maternal mortality surveillance system. For the study period March to August 2020 this data check has been completed.^2^ At hospital discharge 124 pregnancies were ongoing and follow-up information after discharge was obtained for 70/124 women (56.5%). Denominator data were nationwide and from the medical birth registry.

Netherlands

The Netherlands Obstetric Surveillance System (NethOSS) works under the umbrella of the Perined database. Perined is the nationwide perinatal database which contains 98-99% of all pregnancy and birth outcomes in the Netherlands. The perinatal database was established in 1971. National data from 2020 are available (www.peristat.nl). NethOSS sent out weekly reminders to reporting clinicians with a reporting link specific for each clinician in order to register case information about pregnant women with COVID-19.^3^ It was not possible to link NethOSS to the RIVM surveillance registry (national registry for COVID-testing). It was possible to verify the reported information and to add missing information in the primary report by contacting the reporting clinician.

Nordic countries: Denmark, Finland, Iceland, Norway, Sweden

National data were collected in all Nordic countries except Sweden where women from the following areas were included: the University Hospitals at Karolinska, Sahlgrenska and Lund-Malmö, and the regions of Halland, Dalarna and Västmanland.

NOSS Denmark sent bimonthly reminders to clinicians during the study period. NOSS Denmark was linked to the National Microbiology database and the Danish Patient Registry to verify complete reporting, and missing cases were entered retrospectively.^4^ NOSS Finland linked the Finnish Medical Birth Register to the National Infectious Disease Register and the Hospital Discharge Register and could retrieve clinical information from patient records. NOSS Iceland was linked with the Infectious Disease Registry and the National Discharge Database (SAGA). The Medical Birth Registry of Norway sent bimonthly reminders to clinicians during the study period and asked clinicians to confirm the number of new cases or confirm no new cases to report.^5^ Due to legal regulation of registry data use and delays in linkages, it was not possible to link the Medical Birth Registry of Norway with other health registries in Norway for the current report.

In the Nordic countries total number of maternities were retrieved from the Medical Birth Registries based on the following sources:

Denmark total live births (<https://www.dst.dk/en/Statistik/emner/borgere/befolkning/foedsler>)

Finland: total number of births from The Finnish institute of Health and Welfare (<https://thl.fi/en/web/thlfi-en/statistics-and-data/statistics-by-topic/database-reporting>)

Iceland: total number of births from Statistics Iceland (<https://www.statice.is/statistics/population/births-and-deaths/births/>)

Norway: total number of births from The Medical Birth Registry of Norway (<http://statistikkbank.fhi.no/mfr/>)

All the Nordic countries could verify the reported information or retrieve missing information from medical records. National perinatal statistics for 2020 were published in April 2021 in Norway, and perinatal statistics were published between October 2021 and January 2022 for the other Nordic countries. Due to national GDPR regulations numbers less than 3 cannot be reported by country, consequently national datasets are merged in the report. Further details are given in the relevant table footnotes.

UK

UK Obstetric Surveillance System (UKOSS) reporters were requested to report cases daily via a live reporting link and monthly notification reminders were sent to reporting clinicians at all hospitals with an obstetric unit to identify new cases. Following the notification of a case clinicians were reminded to complete the data collection form. Information about maternal or neonatal deaths were cross-checked with the national maternal and perinatal death surveillance, MBRRACE-UK.^6^ UKOSS could not link to other national health registries. For the data used in this analysis missing information about covariates were <5% of cases.

**References**

1. Vandenberghe G, Roelens K, Van Leeuw V, Englert Y, Hanssens M, Verstraelen H. The Belgian Obstetric Surveillance System to monitor severe maternal morbidity. *Facts, Views & Vision in Obgyn* 2017; **9(4):** 181.

2. Donati S, Corsi E, Maraschini A, Salvatore MA and the ItOSS COVID-19 Working Group. The first SARS-CoV-2 wave among pregnant women in Italy: results from a prospective population-based study. *Ann Ist Super Sanità* 2021; **57(4):** 272–85.

3. Overtoom EM, Rosman AN, Zwart JJ, et al. SARS-CoV-2 infection in pregnancy during the first wave of COVID-19 in the Netherlands: a prospective nationwide population-based cohort study (NethOSS). *BJOG* 2022; **129(1):** 91–100.

4. Aabakke AJM, Krebs L, Petersen TG, et al. SARS‐CoV‐2 infection in pregnancy in Denmark—characteristics and outcomes after confirmed infection in pregnancy: A nationwide, prospective, population‐based cohort study. *Acta Obstetricia et Gynecologica Scandinavica* 2021; **100(11):** 2097-110.

5. Engjom H, Aabakke AJM, Klungsøyr K, Svanvik T, Äyräs O, Jonasdottir E, et al. COVID‐19 in pregnancy—characteristics and outcomes of pregnant women admitted to hospital because of SARS‐CoV‐2 infection in the Nordic countries. *Acta Obstetricia et Gynecologica Scandinavica* 2021; **100(9):** 1611-9.

6. Knight M, Bunch K, Vousden N, Morris E, Simpson N, Gale C, et al. Characteristics and outcomes of pregnant women admitted to hospital with confirmed SARS-CoV-2 infection in UK: national population based cohort study. *BMJ* 2020; **369:** m2107.
