# Supplementary material for: Variations across Europe in hospitalization and management of pregnant women with SARS‐CoV‐2 during the initial phase of the pandemic: Multi‐national population‐based cohort study using the International Network of Obstetric Survey Systems (INOSS)
Source: Acta Obstet Gynecol Scand. 2023 Aug 18;102(11):1521–30. doi: 10.1111/aogs.14643 (PMC10577630; doi:10.1111/aogs.14643)
Supplement: Supplementary file 3 — Appendix S3. [file AOGS-102-1521-s004.docx]

**Appendix 3: Details of ethical approval in each INOSS country**

The B.OSS project was approved by the Ethics Committee of the Ghent University Hospital as central Ethics Committee in 2015, and secondary by the EC of every participating hospital (B670201526875).^1^ Women were informed of the study by a paper form, an oral informed consent was required before inclusion.

The Ethics Committee of the Italian National Institute of Health approved the ItOSS project (protocol no. 0010482 CE 01.00, Rome, 24 March 2020) and informed consent was acquired from any woman upon enrolment.

The NethOSS registration system is part of the National Perinatal Registry foundation in the Netherlands (Perined) and did not require specific ethical approval and informed consent of participants.

Ethical approval in Denmark was obtained by the Danish Patient Safety Authority (reg. no. 31-1521-252, 24 April 2020) and the regional Data Protection Agency in Region Zealand (reg. no. REG-022-2020, 23 March 2020). Finland received approval from the Helsinki University hospital (reg. no. HUS1624/2020, 13 May 2020) and from Findata (Dnro THL/5451/14.02.00/2020 June 2^nd^, 2021). Landspitali University Hospital obtained approval from the National Bioethics Committee (reg. no. VSNb2020050016/03.0I, 25 June 2020) and the Icelandic Data Protection Authority (reg. no. 20–106, 9 June 2020). The Norwegian Institute of Public Health, Data, Protection Officer (reg. no. 20_11054, 3 April 2020) and the Western Regional Ethics Committee (reg. no. 125890, 26 March 2020) granted approval in Norway. The Swedish Ethical Review Authority (reg. no. 2020–03012, 11 August 2020 for Sahlgrenska University Hospital and Skåne University Hospital and reg. no. 2020–01499, 22 April 2020 for Karolinska University Hospital). In Denmark, Iceland and Norway, ethical approval exempted the studies from the principle of individual consent. In Finland, individual consent is not required in register-based studies. In Sweden, women received written information about the study, including an opt-out possibility.

The UKOSS study was approved by the HRA NRES Committee East Midlands – Nottingham 1 (ref. no. 12/EM/0365) and individual consent was not required for the collection of pseudo-anonymized routine data.

^1^ Vandenberghe G, Roelens K, Van Leeuw V, Englert Y, Hanssens M, & Verstraelen H. The Belgian Obstetric Surveillance System to monitor severe maternal morbidity. *Facts, Views & Vision in Obgyn* 2017; 9(4): 181.
