# Supplementary material for: Variations across Europe in hospitalization and management of pregnant women with SARS‐CoV‐2 during the initial phase of the pandemic: Multi‐national population‐based cohort study using the International Network of Obstetric Survey Systems (INOSS)
Source: Acta Obstet Gynecol Scand. 2023 Aug 18;102(11):1521–30. doi: 10.1111/aogs.14643 (PMC10577630; doi:10.1111/aogs.14643)
Supplement: Supplementary file 4 — Table S1. Table S2. Table S3. Table S4. Figure S1. Figure S2. [file AOGS-102-1521-s001.docx]

**Table S1.** Neonatal outcomes in births to SARS-CoV-2 PCR positive women admitted to hospital from March to August 2020 by cause of admission and country

|  | **BOSS** | **ItOSS** | **NethOSS** | **NOSS** |  | **UKOSS** |
| --- | --- | --- | --- | --- | --- | --- |
|  | **National, Belgium** | **National, Italy** | **National, Netherlands** | **National,  Denmark, Finland, Iceland & Norway** | **Regional,  Sweden** | **National, United Kingdom** |
| Total births ≥22 (or 24 in UK) weeks of GA *n* |  |  |  |  |  |  |
| COVID-19 related admissions | 35 | 118 | 44 | 23 | 31 | 657 |
| Non-COVID-19 related admissions | 271 | 378 | 48 | 33 | 154 | 504 |
| Stillbirths *n (%)* |  |  |  |  |  |  |
| COVID-19 related admissions | 0 | 1 (0.8) | 0 | 0 | 0 | 4 (0.6) |
| Non-COVID-19 related admissions | 2 (0.7) | 3 (0.8) | 2 (4.2) | 1 (3.0) | 1 (0.7) | 4 (0.8) |
| Neonatal unit admission *n (%)* |  |  |  |  |  |  |
| COVID-19 related admissions | 5 (14.3) | 17 (14.5) | 10 (23.3) | 7 (30.4) | 4 (14.3) | 123 (18.8) |
| Non-COVID-19 related admissions | 32 (12.0) | 51 (13.6) | 7 (15.2) | 5 (15.6) | 17 (11.2) | 48 (9.6) |
| Neonatal death *n (%)* |  |  |  |  |  |  |
| COVID-19 related admissions | 0 | 0 | 0 | 0 | 0 | 2 (0.3) |
| Non-COVID-19 related admissions | 1 (0.4) | 0 | 0 | 0 | 0 | 2 (0.4) |

COVID-19, coronavirus disease 2019; GA, gestational age

**Table S2.** Missing numbers of characteristics and birth outcomes of pregnant women with a positive SARS-CoV-2 PCR test within 7 days prior to admission and up to 2 days after giving birth from March to August 2020 by cause of admission and country

|  | **BOSS** | **ItOSS** | **NethOSS** | **NOSS** | | **UKOSS** |
| --- | --- | --- | --- | --- | --- | --- |
|  | **National, Belgium** | **National, Italy** | **National, Netherlands** | **National,  Denmark, Finland, Iceland & Norway** | **Regional,  Sweden** | **National, United Kingdom** |
| Hospital admission *n* |  |  |  |  |  |  |
| COVID-19 related admissions | 33 | 153 | 45 | 24 | 29 | 656 |
| Non-COVID-19 related admissions | 265 | 386 | 48 | 32 | 151 | 514 |
| Missing Age ≥35 *n (%)* |  |  |  |  |  |  |
| COVID-19 related admissions | 0 | 0 | 0 | 0 | 0 | 0 |
| Non-COVID-19 related admissions | 0 | 7 (1.8) | 0 | 0 | 0 | 0 |
| Missing BMI ≥30 kg/m^2^ *n (%)* |  |  |  |  |  |  |
| COVID-19 related admissions | 1 (3.0) | 2 (1.3) | 7 (15.6) | 0 | 3 (10.3) | 22 (3.4) |
| Non-COVID-19 related admissions | 19 (7.2) | 8 (2.1) | 5 (10.4) | 0 | 0 | 14 (2.7) |
| Missing Migrant background or BAME ethnicity *n (%)* |  |  |  |  |  |  |
| COVID-19 related admissions | 33 (100) | 0 | 5 (11.1) | 0 | 1 (3.4) | 7 (1.1) |
| Non-COVID-19 related admissions | 265 (100) | 0 | 1 (2.1) | 1 (3.1) | 3 (2.0) | 10 (1.9) |
| Missing Parity *n (%)* |  |  |  |  |  |  |
| COVID-19 related admissions | 0 | 1 (0.7) | 0 | 0 | 2 (6.9) | 2 (0.3) |
| Non-COVID-19 related admissions | 0 | 1 (0.3) | 0 | 0 | 0 | 7 (1.4) |
| Missing Gestational age at infection *n (%)* |  |  |  |  |  |  |
| COVID-19 related admissions | 0 | 1 (0.7) | 0 | 1 (4.2) | 5 (17.2) | 0 |
| Non-COVID-19 related admissions | 0 | 12 (3.1) | 0 | 0 | 3 (2.0) | 0 |
| Missing Mode of birth *n (%)* |  |  |  |  |  |  |
| COVID-19 related admissions | 0 | 38 (24.8) | 2 (4.4) | 1 (4.2) | 2 (6.9) | 0 |
| Non-COVID-19 related admissions | 1 (0.4) | 18 (4.7) | 0 | 10 (31.3) | 1 (0.7) | 15 (2.9) |
| Missing Preterm birth <37 weeks *n (%)* |  |  |  |  |  |  |
| COVID-19 related admissions | 0 | 38 (24,8) | 2 (4.4) | 1 (4.2) | 5 (17.2) | 0 |
| Non-COVID-19 related admissions | 1 (0.4) | 30 (7.8) | 0 | 2 (6.3) | 3 (2.0) | 10 (1.9) |

BMI, body mass index; BAME, Black, Asian and minority ethnic; COVID-19, coronavirus disease 2019

**Table S3.** Missing numbers of medical treatment and maternal outcomes among pregnant women admitted due to COVID-19 from March to August 2020 by country

|  | **BOSS** | **ItOSS** | **NethOSS** | **NOSS** | **UKOSS** |
| --- | --- | --- | --- | --- | --- |
|  | **National,**  **Belgium** | **National,**  **Italy** | **National, Netherlands** | **National,  Denmark, Finland & Norway** | **National,  United Kingdom** |
| Total women admitted due to COVID-19 *n* | 33 | 153 | 45 | 24 | 656 |
| Medical treatment *n (%)* |  |  |  |  |  |
| Missing antibiotics (alone) | 0 | 1 (0.7) | 0 | 2 (8.3) | 0 |
| Missing antivirals (alone) | 0 | 1 (0.7) | 0 | 2 (8.3) | 0 |
| Missing antibiotic and antiviral in combination | 0 | 1 (0.7) | 0 | 2 (8.3) | 0 |
| Missing hydroxychloroquine | 0 | 1 (0.7) | 0 | 2 (8.3) | 0 |
| Missing anti Interleukin 6 | 0 | 153 (100) | 0 | 2 (8.3) | 0 |
| Missing Iv Immunoglobulins | 0 | 153 (100) | 45 (100) | 2 (8.3) | 0 |
| Missing steroids for fetal indication | 0 | 1 (0.7) | 0 | 2 (8.3) | 0 |
| Missing steroids for maternal indication | 0 | 153 (100) | 0 | 2 (8.3) | 0 |
| Missing thrombosis prophylaxis | 0 | 1 (0.7) | 45 (100) | 2 (8.3) | 0 |
| Missing anti-thrombotic treatment | 0 | 153 (100) | 45 (100) | 2 (8.3) | 0 |
| Missing ICU admission *n (%)* | 0 | 0 | 0 | 1 (4.2) | 0 |
| Maximum level of respiratory support *n (%)*^*^ |  |  |  |  |  |
| Missing mechanical ventilation or ECMO | 0 | 0 | 0 | 1 (4.2) | 57 (30.0) |
| Missing CPAP or high flow nasal cannula | 33 (100) | 0 | 45 (100) | 10 (41.7) | 57 (30.0) |
| Missing oxygen supplementation | 33 (100) | 0 | 0 | 3 (12.5) | 57 (30.0) |
| Missing maternal death *n (%)* | 0 | 0 | 0 | 0 | 0 |

COVID-19, coronavirus disease 2019; CPAP, continuous positive airway pressure; ECMO, extracorporeal membrane oxygenation; ICU, intensive care unit; Iv, intravenous; ^*^In UKOSS, maximum level of respiratory support measured only among women who received respiratory support (n=190); level of respiratory support unknown for 57/190 women

**Table S4.** Missing numbers of neonatal outcomes in births to SARS-CoV-2 PCR positive women admitted to hospital from February to August 2020, by cause of admission and country

|  | **BOSS** | **ItOSS** | **NethOSS** | **NOSS** |  | **UKOSS** |
| --- | --- | --- | --- | --- | --- | --- |
|  | **National, Belgium** | **National, Italy** | **National, Netherlands** | **National,  Denmark, Finland, Iceland & Norway** | **Regional,  Sweden** | **National, United Kingdom** |
| **Neonatal outcomes** |  |  |  |  |  |  |
| Total births ≥22 (or 24 in UK) weeks of GA *n* |  |  |  |  |  |  |
| COVID-19 related admissions | 35 | 118 | 44 | 23 | 31 | 657 |
| Non-COVID-19 related admissions | 271 | 378 | 48 | 33 | 154 | 504 |
| Missing Stillbirths *n (%)* |  |  |  |  |  |  |
| COVID-19 related admissions | 0 | 0 | 0 | 0 | 3 (9.7) | 0 |
| Non-COVID-19 related admissions | 2 (0.7) | 0 | 0 | 0 | 2 (1.3) | 0 |
| Missing Neonatal unit admission *n (%)* |  |  |  |  |  |  |
| COVID-19 related admissions | 0 | 0 | 1 (2.3) | 0 | 3 (9.7) | 4 (0.6) |
| Non-COVID-19 related admissions | 2 (0.7) | 0 | 0 | 0 | 2 (1.3) | 4 (0.8) |
| Missing Neonatal death *n (%)* |  |  |  |  |  |  |
| COVID-19 related admissions | 0 | 0 | 0 | 0 | 0 | 3 (0.5) |
| Non-COVID-19 related admissions | 4 (1.5) | 0 | 0 | 0 | 0 | 2 (0.4) |

COVID-19, coronavirus disease 2019; GA, gestational age

**Figure S1.** Flowchart describing the study population


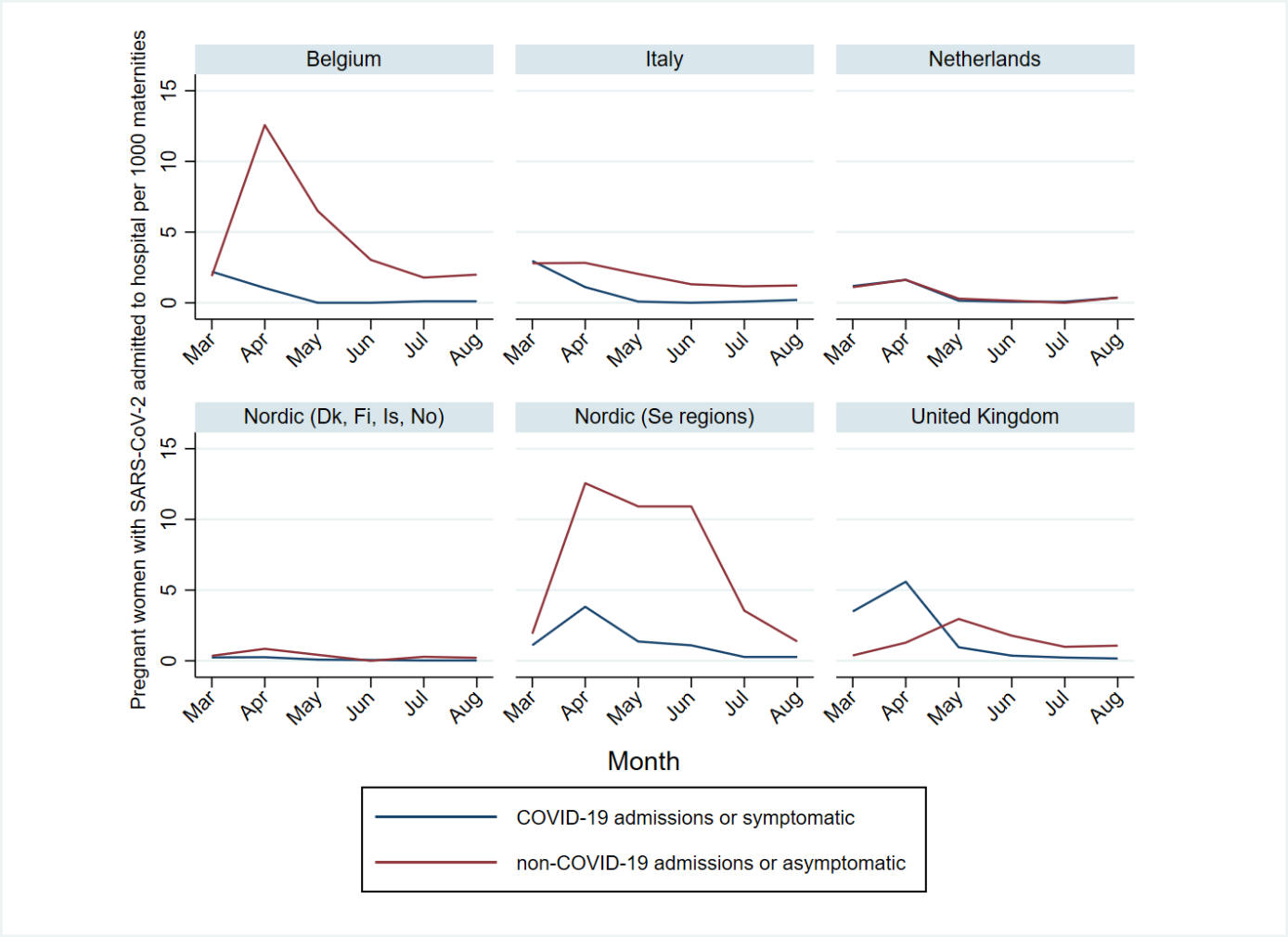


**Figure S2.** Pregnant women with SARS-CoV-2 admitted to hospital per 1000 maternities per month of first positive PCR test, March to August 2020, by country and admission reason
